# Vowel- and Diphthong-Like Spectral Patterns in Sperm Whale Codas

**DOI:** 10.1162/OPMI.a.252

**Published:** 2025-11-02

**Authors:** Gašper Beguš, Ronald L. Sprouse, Andrej Leban, Miles Silva, Shane Gero

**Affiliations:** Department of Linguistics, University of California, Berkeley, Berkeley, CA, USA; Department of Statistics, University of Michigan, Ann Arbor, MI, USA; Department of Brain and Cognitive Sciences, Massachusetts Institute of Technology, Cambridge, MA, USA; Department of Biology, Carleton University, Ottawa, ON, Canada; The Dominica Sperm Whale Project, Roseau, Dominica; Project CETI, New York, NY, USA and Roseau, Dominica

**Keywords:** communication, marine biology, linguistics, cognitive science, sperm whales, phonology

## Abstract

The sperm whale communication system, consisting of groups of clicks called *codas*, has been primarily analyzed in terms of the number of clicks and their inter-click timing. This paper reports spectral properties in sperm whale vocalizations and demonstrates that spectral properties are highly structured, discretely distributed across codas, and uttered in dialogues, rather than being a physical artefact of whale movement. We report formant structure in whale codas and uncover previously unobserved spectral patterns. We argue that these spectral properties freely combine with the traditionally analyzed properties. We present a visualization technique that allows the description of several previously unobserved patterns. Codas are on many levels analogous to human vowels and diphthongs and can be conceptualized in terms of the source-filter theory: vowel duration and pitch correspond to the number of clicks and their timing (traditional coda types), while spectral properties of clicks correspond to formants in human vowels. We identify two recurrent and discrete coda-level spectral patterns that appear across individual sperm whales and across traditional coda types: the *a*- and *i*-coda vowels. We also report that sperm whales have diphthongal patterns on individual codas: with rising, falling, rising-falling and falling-rising formant patterns observed. These uncovered patterns suggest that spectral properties have the potential to add to the communicative complexity of codas independent of the traditionally analyzed properties and add a new dimension to the study of a cetacean communication system.

## INTRODUCTION

Sperm whale vocalizations are among the most intriguing communication systems in the animal kingdom. How sperm whales (*Physeter macrocephalus*) might encode information into their communication system is one of the big open questions in animal research. Sperm whales communicate with click vocalizations that they group into units called *codas* (Watkins & Schevill, 1977; Whitehead, 2003; Whitehead & Weilgart, 1991; Worthington & Schevill, 1957). Clicks in such codas are acoustically distinguishable from echolocation clicks (Madsen, Wahlberg, & Møhl, 2002; Madsen, Payne, et al., 2002; Møhl et al., 2003). Coda vocalizations are likely culturally learned (Rendell et al., 2012; Rendell & Whitehead, 2003), and different culturally defined clans feature different coda types (Amano et al., 2014; Amorim et al., 2020; Andreas et al., 2022; Gero, Whitehead, & Rendell, 2016; Huijser et al., 2020; Rendell & Whitehead, 2003). Many coda types have been identified based on two primary characteristics: the number of clicks and the timing between clicks (Weilgart & Whitehead, 1993).

The communication system of sperm whales has thus far been analyzed primarily as a binary system. Nearly all research on sperm whale codas focuses primarily on the number of clicks and their timing, but not their spectral properties. The two properties—the number of clicks and their timing—are used to classify codas into groups traditionally called coda (Weilgart & Whitehead, 1993; Whitehead, 2003). Recently, Sharma et al. (2024) demonstrated that these traditionally analyzed properties can be further analyzed as four distinct timing/click number features (“rhythm, tempo, rubato, and ornamentation”). Because our proposal focuses on a different dimension—spectral properties rather than timing/click number features—we use the traditional coda type notation throughout the paper.

This paper proposes that another dimension—spectral properties of clicks—are highly structured, discretely distributed among codas, and thus potentially meaningful in the communication system of sperm whales. We hypothesize that recurrent spectral patterns can be observed across individual whales. We report formants (or resonant frequencies) in codas and describe and quantify these patterns, raising the possibility that sperm whales actively control spectral properties which have the potential to carry meaning.

For this purpose, we apply the source-filter theory of speech production as it is used to describe human vowels, which have a quasiperiodic glottal source filtered by the supraglottal vocal tract (Fant, 1971; Stevens, 1999). Sperm whales vocalize by sending air through the phonic lips which vibrate and result in pulses often called “clicks” in sperm whales (Huggenberger et al., 2016; Madsen et al., 2023; Madsen, Payne, et al., 2002). Humans produce vowels such as [a], [i] or [u] by letting air through the vocal folds, which vibrate and result in vocalic pulses. Under our proposed framework, whale clicks are analogous to the pulses of vocal folds in human speech production. In other words, we treat vibrations of the phonic lips as the source in the source-filter theory approach.

We note there is a potential terminological confusion regarding “click”, which has a different meaning in the description of human speech that is not analogous to a sperm whale click. In human phonetics, a “click” is a consonant created by a compound closure of the oral cavity with a release that results in a non-pulmonic airstream and ingressive turbulent airflow (Ladefoged & Maddieson, 1996). The aperiodic noise of the turbulence is its characteristic source in the source-filter model and is distinct from the quasiperiodic glottal source of a vowel. While individual sperm whale clicks have some acoustic similarity to the influx phase of human click consonants, particularly in their short duration and rapid rise and fall of the acoustic signal, they are not similar in articulation and do not contain turbulent airflow. Sperm whale clicks are better conceived as pulses akin to the glottalic pulses of human vowel production. Both are vibrations resulting from airflow and appear in higher-level structures of quasiperiodic sequences.

**Figure 1.**
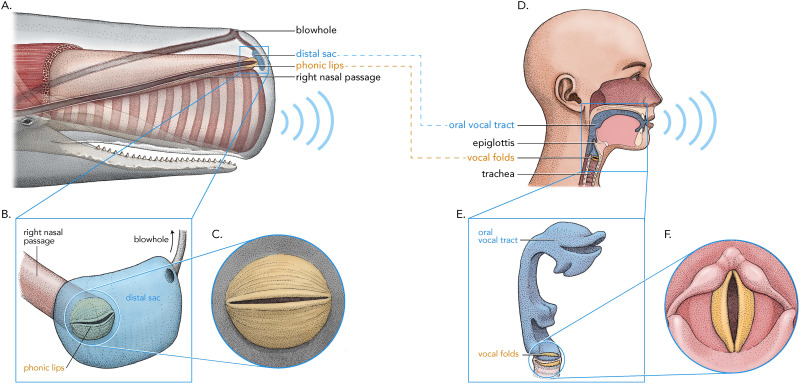
Parallels in human vowel (D) and sperm whale coda (A) vowel production. The source articulators, the phonic lips (C) correspond to human vocal folds (F). We hypothesize that the filter articulators, the distal air sac (B), corresponds to the vocal tract (E). CC 4.0 BY © Alex Boersma.

**Figure 2.**
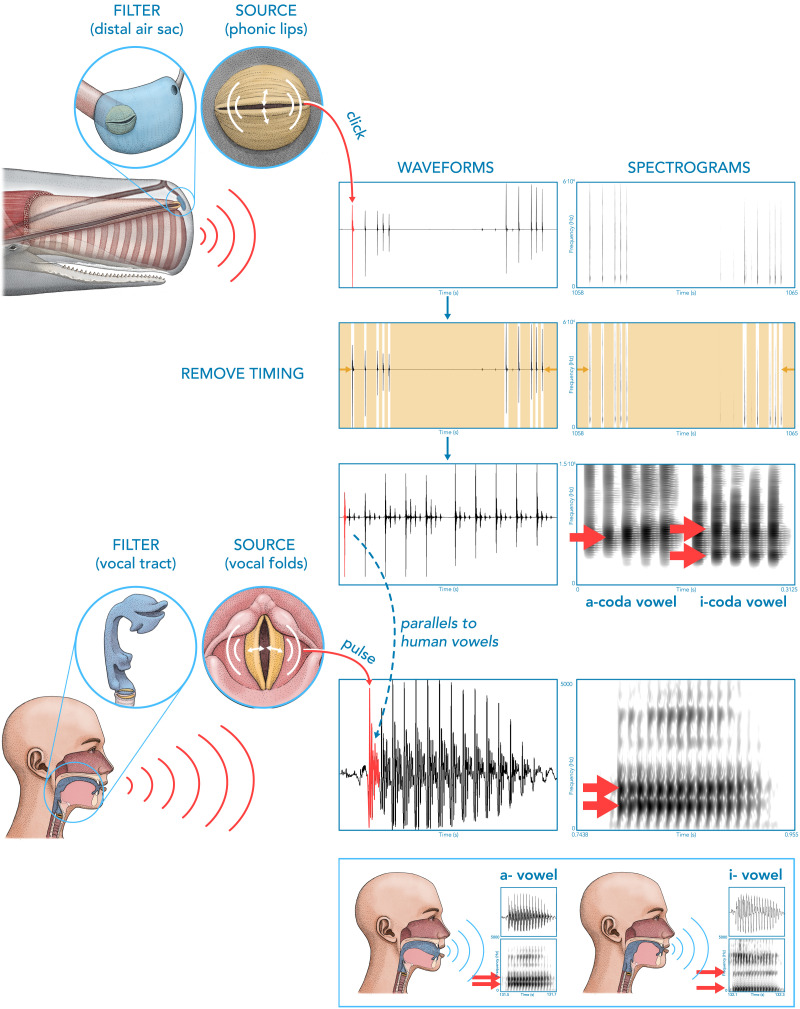
An illustration of the parallels between the production and acoustics of human vowels compared with sperm whale coda-vowels. Each open-close cycle of the source articulators (vocal folds and phonic lips) produces a vocalic pulse (color-coded in red). The shape of the articulatory filter (vocal tract or distal air sac) results in formant structures with a high degree of similarity between humans and sperm whales when timing is removed (color-coded in yellow). (**top left**) A waveform and a spectrogram (0–60 kHz) of two consecutive focal codas by Pinchy (6931 and 6932) when timing is not removed. (**top right**) Spectrograms (0–15 kHz) of the same two codas using our visualization approach. This visualization uncovers a clear pattern: the first coda features a single formant (is of the *a*-coda vowel type), the second has two formants (is of the *i*-coda vowel type). (**bottom**) A waveform and a spectrogram (0–5000 Hz) of a vowel [a] and [i] produced by one of the authors in Slovenian, illustrating how the change of the shape of the vocal tract in humans results in different formant frequencies (red arrows). Illustrations CC 4.0 BY © Alex Boersma.

Vibrations of human vocalic pulses travel through the vocal tract (or filter). Based on the shape of the vocal tract, different resonant frequencies (formants; for terminology, see Titze et al., 2015) form in human vowels, which cause the differences between human vowels such as [a], [i] or [u]. For example, a low tongue placement results in a vocal tract shape that produces the vowel [a] with formants close together, whereas a high tongue placement results in the human vowel [i] with formants placed wider apart (Figure 2). Formants are visible in spectrograms in Figure 2 as shaded areas and marked with red arrows throughout this paper. We hypothesize that the distal air sac in sperm whales similarly acts as a resonant body (filter) that modulates resonant frequencies (Figure 1). Like in humans where the vocal tract begins with the vocal folds, the phonic lips in sperm whales are located at the entrance to the distal air sac. We show that codas feature two discrete patterns in their resonant frequencies that match formants in human speech. We show that these patterns are recurrent across different whales. In this paper, we use *spectral peaks* when we refer to peaks in the FFT spectrum of individual clicks (or pulses). We use *formants* to describe resonant frequency contours at the entire coda level as calculated by LPC formant analysis (see section Formant Analysis for details).

Under this approach, the *coda types* correspond to human vowel duration (the number of pulses/clicks) and pitch (F0) in human speech. Recurrent spectral properties observed in whale codas (that we call *coda vowels*) correspond to formant frequencies, i.e., vowel identity in human speech (Table 1). Coda diphthongs are those vocalic patterns in which formants are not flat through a coda vocalization, but show upward, downward, or other trajectories. Pitch (F0) in humans is to a large degree orthogonal to vowel quality. For example, in tonal languages such as Mandarin Chinese, syllables with vowels [a] or [i] can feature all four tones (Duanmu, 2007). We hypothesize that spectral patterns in sperm whales (called *coda vowels*) are similarly orthogonal to the coda type: the described vocalic and diphthongal patterns are possible on different coda types.

**Table 1.** Parallels in human vowels and sperm whale codas. The table distinguishes the source features and filter features according to the source-filter theory of speech production.

	**Human vowels**	**Sperm whale codas**
**source** (vocal folds/phonic lips)	vowel duration	number of clicks
	F0	inter-click interval (ICI)
**filter** (vocal tract/distal air sac)	height & backness (F1 & F2)	coda vowel (*a* vs. *i*)
	formant trajectories	diphthongal codas

### Prior Work

Acoustic studies of sperm whale clicks have included inter-pulse and inter-click analyses (Oliveira et al., 2016) as well as spectral analyses (Moore et al., 1993; Thode et al., 2002; Whitehead, 2003), but no recurrent coda-level patterns that might be meaningful has been previously observed in the spectral domain. That some clicks can have multiple peaks which shift with depth has been observed in Thode et al. (2002), Goold and Jones (1995), and Lin et al. (2017) also report one or two peaks, Huggenberger et al. (2016) reports one. Thode et al. (2002) conclude that these shifting peaks might be at least partly controlled by the whales, but the multiple peaks that shift with depth are analyzed primarily as a feature of movement (how deep the whales dived), which is contrary to what we claim here. Thode et al. (2002) only report acoustic properties of echolocation clicks, which serve a different purpose from codas. None of the prior works report the existence of coda-level formants (resonant frequencies) or any patterns at the coda level that would not be movement-related and discretely distributed at the coda-level (rather than at the click-level) across whales.

Other odontocetes seem to primarily modulate the fundamental frequency (F0), i.e., the frequency of their phonic lip vibration when communicating. This is primarily obvious in whistle-like vocalizations of dolphins, orcas, or beluga whales (Filatova et al., 2015; McCowan & Reiss, 1997, 2001; Panova et al., 2016, 2019). Even pulsed call vocalizations seemed to primarily be modulated in the fundamental frequency (F0) (Sportelli, 2019; Wellard et al., 2020). The same is true for vocalizations that Panova et al. (2016) call “vowel”-like based on an acoustic impression. The work on orcas, dolphins, or beluga whales does not describe spectral patterns that would be independent of the fundamental frequency (= formants that originate from manipulating the filter part in the articulatory process) and discretely distributed in types as is the case in sperm whales. Vocalizations of odontocetes are not usually analyzed in terms of source filter theory or in terms of analogs between human vowels and whale vocalizations except when studying acoustic correlates of whale size (Samarra & Miller, 2006) or based on impressionistic similarity (Panova et al., 2016). To our knowledge, no reports exist that odontocetes modulate resonant frequencies which would result in simple discretely distributed patterns that are discoverable without dimensionality reduction techniques and at least partially orthogonal in terms of the *source* features (such as F0) and *filter* features (such as formant modulation).

Humpback whale (*Megaptera novaeangliae*) vocalizations have been compared to human vowels (or speech; Kello et al., 2017) and language’s distributional laws (Arnon et al., 2025), but the described patterns require clustering techniques (Pines, 2019) and are not discretely distributed. Additionally, the primary function of humpback song is likely mating or male collaboration (Darling et al., 2006; Herman, 2017; Whitehead & Rendell, 2014), which stands in contrast to sperm whale and other odontocetes where vocalizations likely have a non-mating social communicative function (Weilgart & Whitehead, 1993). To be sure, other animals are able to modulate formant frequencies both in their vocalizations in the wild (Fitch, 2000; Fitch & Hauser, 2003; Stansbury & Janik, 2019; Stoeger et al., 2012) and when imitating human vocalizations (such as gray seals, parrots, elephants or chimpanzees; Duengen et al., 2023; Ekström et al., 2024; Klatt & Stefanski, 1974; Patterson & Pepperberg, 1998; Stansbury & Janik, 2019; Stoeger et al., 2012).

### Spectral Properties

We propose a new visualization technique for codas (Figure 2) that effectively removes timing and allows us to observe previously unreported spectral patterns at the coda level. We describe the *a*-coda vowel and the *i*-coda vowel as well as diphthongal patterns. To confirm these patterns, we perform automatic quantitative analyses of coda vowels where we employ the same methodology as is standard in human acoustic research on vowels: linear predictive coding (LPC). We also manually annotate all codas in our data set to compare the automatic and manual analyses.

That spectral properties might be meaningful in the sperm whale communication system has been recently proposed in Beguš, Leban, and Gero (2023). A fiwGAN model (Beguš, 2021a) was trained to imitate sperm whale codas and to embed information into the learned vocalizations. Building on Beguš (2020), an introspection technique to test for the meaningful properties a model learns from unknown data was developed and applied to sperm whale communication in Beguš, Leban, and Gero (2023). The model learned properties previously considered potentially meaningful: the number of clicks and their inter-click intervals. The model network also learned acoustic properties that previously were not considered potentially meaningful: spectral mean and acoustic regularity.

While the interpretability technique in Beguš, Leban, and Gero (2023) pointed to candidate properties learned as meaningful by the models, here we explicitly uncover and describe the spectral patterns. In other words, AI models have been shown to be useful as hypothesis space reduction techniques (Andreas et al., 2022; Davies et al., 2021; Goldwasser et al., 2023; Jumper et al., 2021; Stokes et al., 2020) that can offer clues to researchers, but do not yet provide the explicit mechanisms. In our case, the interpretability technique uncovered that a part of the network that generates data considered spectral properties to be meaningful. This present work is thus a post hoc explicit analysis of a clue provided by fiwGAN (Beguš, 2021a) and an interpretability technique (Beguš, Leban, & Gero, 2023). We explicitly describe several acoustic patterns that we observe during the acoustic analysis and posit that they might be meaningful in sperm whale vocalizations. Findings of this paper thus suggest that the approach proposed in Beguš, Leban, and Gero (2023) where a fiwGAN is trained on the unknown data and the *causal disentanglement with extreme values* (CDEV; Beguš, Leban, & Gero, 2023) technique is applied on learned representations can be successful for uncovering potentially meaningful properties in unknown communication systems.

### Terminology

Our proposed terminology is analogical and not taxonomic. We introduce the labels *coda vowel* and *coda diphthong* to describe the newly observed patterns in sperm whale codas within the source-filter framework. The “vowel” and “diphthong” parts denote the acoustic analogues to human vowels, whereas the modifier “coda” signals differences between human vowels, sperm whale coda vowels and other animal vocalizations. We take the presence of formant patterns with discretely distributed and controlled types that interact with the source features such as duration and F0 as sufficient conditions to term vocalizations vowel-like.

A key distinction remains: human vowels are phonemic, which means they distinguish meaning. No referential meaning relationship has yet been established for sperm whale codas. While it is possible or even likely that codas do distinguish or carry referential meaning, this has not yet been observed. In this respect, the coda vowels are an observed pattern that does not have an established function yet. It is also possible that the described patterns are not used by whales to express referential meaning, but are in some other non-meaningful function or a consequence of some currently not understood mechanism. One challenge in this line of inquiry is the relative difficulty of observing and recording the complex behavior of sperm whales. Despite the differences between coda vowels and human vowels, conceptualizing sperm whale codas as analogical to vowels is justified by the common mechanisms in both the production and the acoustics of the two systems as well as highly practical for representational purposes (e.g., for transcribing dialogues in Tables 3 and S1). Additionally, while referential meaning has not been established for codas, the social meaning of codas is well documented (Gero, Whitehead, & Rendell, 2016; Hersh et al., 2022; Rendell & Whitehead, 2003). Sperm whales use different coda types to signal clan identity (Gero, Whitehead, & Rendell, 2016; Hersh et al., 2022; Rendell & Whitehead, 2003). Treating coda vowels as parallel to human vowel production is thus additionally warranted, as we know that human vowel realization carries social meaning in addition to distinguishing referential meaning (Eckert & Labov, 2017). As Eckert and Labov (2017) point out, for example, “[i]n English, continuous variation in the phonetic realization of vowel allophones is the most heavily employed resource for the construction of social meaning”.

## RESULTS

### Vowels

We performed an acoustic analysis of clicks produced by the whales who were wearing a tag (focal clicks). We use the LPC algorithm as well as simple local maxima algorithms on spectra to find formants or peaks (for details, see Methods section). To estimate the coda-level spectral properties, we performed formant analysis based on linear predictive coding (LPC), which is a standard method for analyzing formants in human vowels. We assigned codas into *a*-type and *i*-type categories based on the number of formants detected in each coda and then compared the results with the categories assigned by human annotation. There were 1,375 focal codas in the dataset. We excluded a total of 161 codas from analysis because they were associated with an unidentified whale or were produced by one of three whales that had few codas overall and no *i*-type coda vowels, which makes it difficult to observe the coda vowel pattern (unknown *n* = 108, LADYO *n* = 7, NALGENE *n* = 12, SOURSOP *n* = 34). After exclusions, a total of 1,214 analyzed codas were included in the analysis. For acoustic specification of the tags used for recordings (the Dtag), see Supplementary Materials.

**Figure 3.**
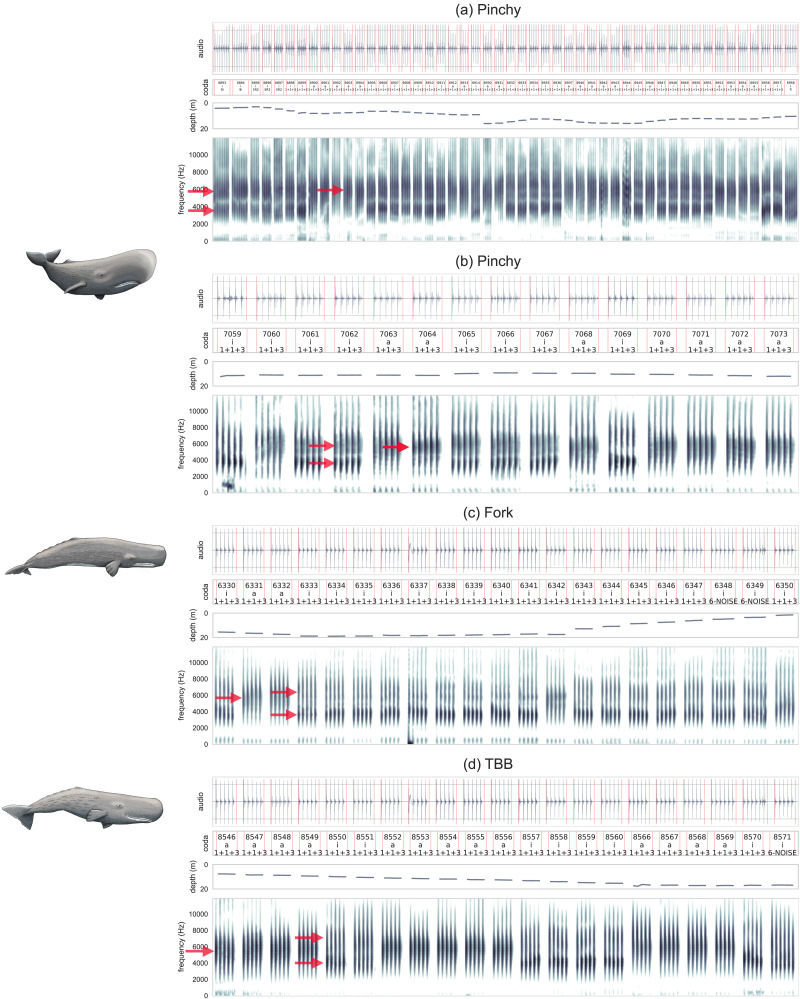
Waveforms and spectrograms (0–12,000 Hz, single channel) of *a*- and *i*-coda vowels along with traditional coda classification from focal Pinchy (top two figures), Fork (middle), and TBB (bottom). A few formants are annotated with red arrows, where *a*-coda vowels have a single formant and *i*-coda vowels have two. Timing has been removed according to the described technique and all clicks peak-normalized. Pinchy’s and TBB’s codas are from two consecutive bouts (the second bout starts with coda 7065 for Pinchy and coda 8566 for TBB). Depth values (in m) for each coda are below each spectrogram. Illustrations CC 4.0 BY © Alex Boersma.

Based on the quantitative analysis, we report a newly observed coda-level pattern: there are at least two distinct spectral patterns in sperm whale codas across studied whales. One group consists of codas with a single pronounced formant below 10 kHz (at 5,780 Hz). The other group consists of codas with two formants below 10 kHz (at 3,757 Hz and 6,579 Hz). We term the first pattern the “*a*-coda vowel” and the latter the “*i*-coda vowel” based on the analogy with human vowels where the first and second formants in the [i] vowel are farther apart from the formants in [a] vowels. Human vowels [a] and [i] also differ from our *a*- and *i*-coda vowel in many ways (e.g., the exact values and number of formants), but based on the aforementioned analogues and utility of using letters to distinguish coda vowel types, we propose the *a*-coda vowel and *i*-coda vowel notation. Figure 2 illustrates the distinction between two coda vowels. Crucially, as is clear from the spectrograms of several codas by Pinchy, Fork, and TBB in Figure 3, these coda vowels are discrete: a coda is either of type *a* or type *i*. In other words, the coda vowel features are discretely distributed across codas.

That coda vowels are probably actively controlled by whales is suggested by the fact that there is little mixing between one and the other spectral pattern within codas. In other words, the coda vowel types are discretely distributed across codas: if a coda is of the *a*-type, all clicks will feature a single formant and vice versa; if a coda is of the *i*-type, all clicks have two formants. If the distinction were random or an automatic consequence of some external factor, we would expect mixing between *a*-like and *i*-like clicks. Figure 3 shows that all clicks within a given coda either are of the *a*-coda vowel type (have one spectral peak or formant) or *i*-vowel type (have two spectral peaks or formants), but these can change between subsequent codas in an exchange.

To quantitatively confirm that the patterns are in the majority of cases discrete, we performed Fourier analysis on each click separately. We used *scipy* (Virtanen et al., 2020)’s *welch* function to estimate spectral power in a 3.5 ms audio segment of each click’s first pulse. The segment included the 2.0 ms preceding the click peak, which was sufficient to include the signal rise to the peak without interference from preceding clicks. The mean time between the first and second click pulses for all focal clicks was 3.1 ms, and 1.5 ms after the click peak was selected to include approximately the first half of the inter-pulse interval. The *scipy find_peaks* function located peaks in the calculated spectrum between 1,000 and 10,000 Hz. The minimum distance between adjacent peaks was 1,500 Hz, and the minimum height of each peak was defined as 25% of the highest peak in the range. The frequencies of the two highest spectral peaks returned by *find_peaks* were selected and labeled in order as the first and second spectral peaks.

**Figure 4.**
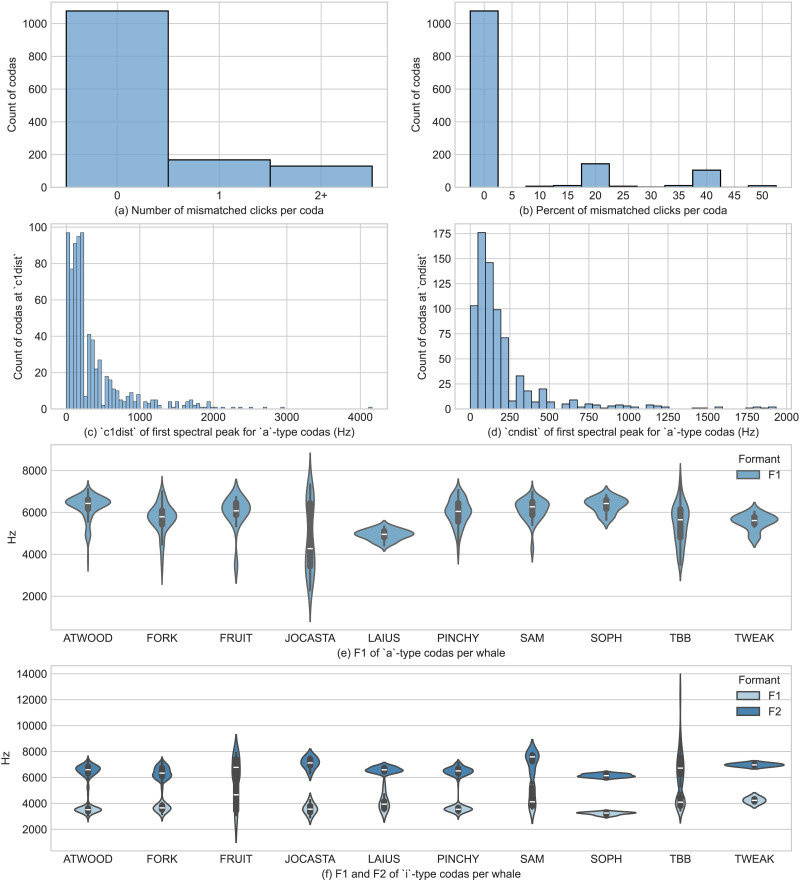
(**top**) The number (left) and percent (right) of mismatched clicks per coda. (**middle**) The mean distances in Hz between (left) the spectral peaks of each non-initial click from the corresponding peak in the first click (*c1dist*); and (right) of the spectral peaks of each non-initial click from the preceding click (*cndist*). (**bottom**) Violin plots of the full distributions of F1 (e, f) and F2 (f) for ten whales, with inner box plots displaying the median and interquartile range, show a high degree of consistency across all analyzed whales.

We then assigned *a*- and *i*-type labels to each click based on the spectral peaks. Clicks for which two or more spectral peaks were found between 1,000 and 10,000 Hz were classified as *i*-type, and clicks with fewer peaks were classified as *a*-type. A click was considered to be mismatched if it did not receive the same classification as the majority of clicks in the coda. The results in Figure 4a show the number of mismatched codas based on this classification. It demonstrates that in the majority of codas, all clicks are of the same type. Approximately 78% of codas (*N* = 1,077) contain no mismatched clicks. In approximately 12% of codas (*N* = 168), only one click is mismatched. Codas with two or more mismatched clicks form the smallest group of approximately 9% (*N* = 130).

To estimate mean values of F1 and F2, we performed LPC analysis on 724 focal *a*-coda vowels and 397 focal *i*-coda vowels, using the hyperparameters described in Data section. The percentages of coda vowel classifications produced by the algorithm that matched the hand label were calculated for each set of hyperparameters. We selected the set(s) of parameters for each whale that best matched the hand-labeled vowel annotations and used the formant analysis produced by those parameters to calculate formant means (Table 2) for each vowel type.

**Table 2.** Counts of hand-labeled focal *a*- and *i*-type vowels for each whale. The formant analysis using each hyperparameter set assigned *a* and *i* labels that were compared with hand-labeled types. The % Agree value shows the percentage of matches with the hand-labeled types for the best performing parameter set(s) for the whale. The psetsN value indicates the number of hyperparameter sets that produced the best performance. The mean formant values produced by the best performing parameter sets are shown in Hz as f1/f2.

whale	psetsN	% Agree	focal *a*		focal *i*		
			count	f1	count	f1	f2
ATWOOD	1	90.3	208	6266	151	3586	6563
FORK	1	80.9	182	5738	111	3830	6414
FRUIT	3	90.6	26	5899	6	4703	7046
JOCASTA	1	98.2	48	4790	8	3568	7192
LAIUS	9	89.7	20	4938	9	4054	6557
PINCHY	2	89.3	78	6015	62	3649	6497
SAM	14	94.2	47	6089	5	4391	7311
SOPH	99	100.0	14	6233	14	3253	6148
TBB	2	79.5	88	5464	29	4386	7290
TWEAK	84	100.0	13	5324	2	4255	7011

The observed pattern is not an idiosyncratic property of a single whale, but a recurrent feature across whales. Figure 3 shows the raw spectrograms of the *a*-/*i*- interchange pattern on three different whales within the same bout and the same 1 + 1 + 3 coda type (a bout is a set of codas where timing between two codas is not greater than 10 s). The violin plots in Figure 4e, f summarize the median, interquartile range, and full distribution of each coda’s F1 and F2 of the two coda vowels and show a high degree of consistency across all analyzed whales. This consistency is found in both the *a*- and *i*-coda vowels and is highly uniform across the ten whales. There are a total of 14 focal whales in our data (Sally is excluded because she was recorded together with TBB), but some whales have only a few focal codas recorded. Lady Oracle has only 7 codas, Nalgene only 12, and Soursop a total of 34. In these whales the *i*-coda vowel is missing, but it is difficult to establish whether these whales lack the *i*-coda vowel pattern completely or whether there is an accidental gap due to data sparsity or noise in the recordings. As a result these whales were excluded from the analysis. Atwood, Fork, Pinchy, and TBB represent over 2/3 of all analyzed clicks in the corpus; the *a*-/*i*-coda vowels are found in all these well-represented whales.

Both coda vowels occur on several traditional coda types. One of the most frequent coda types in the Eastern Caribbean Clan is the 1 + 1 + 3 coda, which is a coda with five clicks with the first two inter-click intervals (ICIs) long, followed by two short ICIs. Other frequent types include 5R1 and 5R2 codas which contain 5 clicks regularly (R) spaced. The first digit in the traditional coda classification denotes the number of clicks in a coda (e.g., 5, 6, 7, etc.). The following letter optionally describes the timing between clicks: regular (R), decreasing (D) or increasing (i) ICI timing. The ‘+’ symbol denotes an increased duration between clicks (hence 1 + 1 + 3) (Weilgart & Whitehead, 1993; Whitehead, 2003). The digit after the type denotes coda duration: 1 stands for shorter duration, 2 for longer duration (Gero, Whitehead, & Rendell, 2016).

Figure 3 shows an interchange between *a*- and *i*-coda vowels on the 1 + 1 + 3 coda type. Figure S8 (Supplementary Materials) shows three cases of 5R2 coda types with the *i*-coda vowel pattern by Pinchy and the same coda type with the *a*-coda vowel pattern by Fork. The exchange between the two vowels can happen within the same bout. In other words, nearly all coda types in these figures are of the 1 + 1 + 3 type. This is in part the result of the 1 + 1 + 3 coda type dominating the coda type production repertoires for whales from the Eastern Caribbean Clan (Gero, Bøttcher, et al., 2016). Nevertheless, the seemingly repeated 1 + 1 + 3 codas show a high degree of variability between the *a* and the *i* codas.

### Diphthongs

In addition to the two patterns (*a*- vs. *i*-coda vowel), we observe that some codas feature upwards, downwards or other types of trajectories in formant frequencies. Diphthongal patterns are rarer in our data compared to level codas (as is clear from the spectral differences analysis in Figure 4). Diphthongs fall in a distinct spectral category and we present additional evidence that the observed spectral patterns are probably controlled by whales and are not artefacts, as we show that formant trajectories do not automatically follow from whale movement or depth.

**Figure 5.**
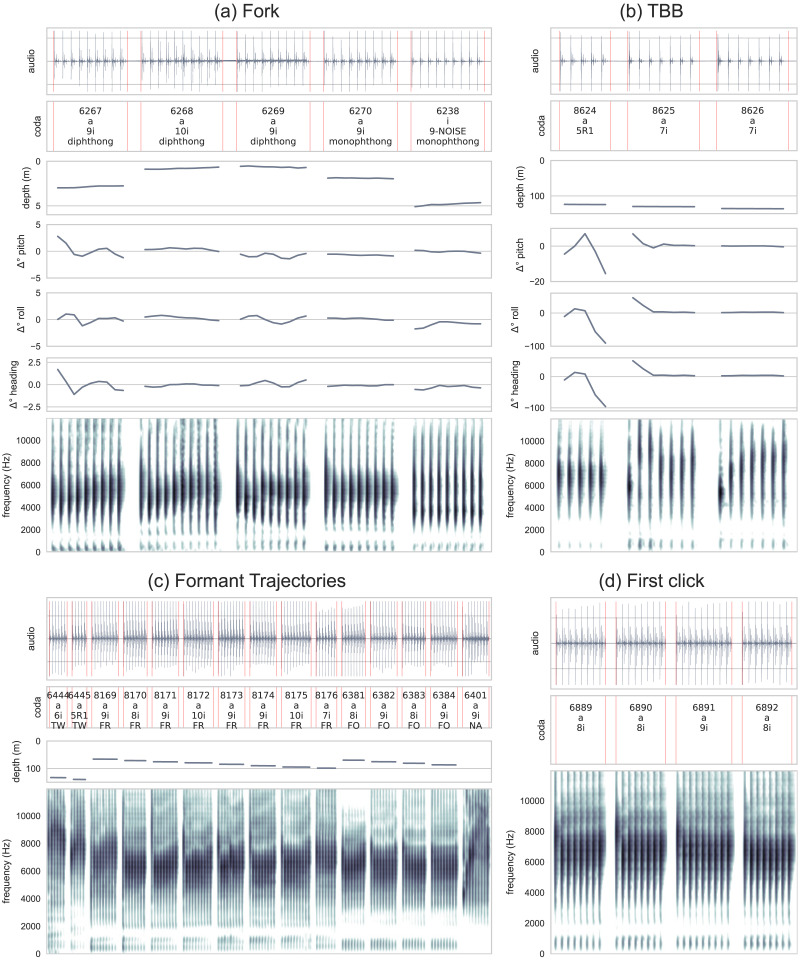
(**top left**) Waveforms and spectrograms 0–12,000 Hz (single channel) from four consecutive codas and an additional coda (rightmost) recorded from a tag on Fork. The first three figures from the left illustrate diphthongal patterns (the first two codas exhibit an upward trajectory, the third coda a downward trajectory); the two figures on the right illustrate level formants. The codas are of the 9i, 10i, 9i, 9i, and 9i type. The first four codas are of the *a*-type, the last one of the *i*-type. All codas are from the same bout. Fork’s vocalizations are accompanied by corresponding depth and Δ° of head, pitch, and roll. (**top right**) Waveforms and spectrograms 0–12,000 Hz (single channel) from three consecutive codas recorded from a tag on TBB. The codas are of the 5R1, 7i, and 7i type and *a*-coda vowel type. The first coda shows a level trajectory, the second coda a falling trajectory, and the third coda a rising trajectory. All codas are from the same bout with corresponding position data for the observed codas: depth, head, pitch, and roll. (**bottom left**) Waveforms and spectrograms (0–12,000 Hz; single channel) of diphthongs with substantial formant trajectories from four different whales with depth values (in m) for each corresponding coda. (**bottom right**) Waveforms and spectrograms (0–12,000 Hz, single channel) of codas from Fork (single channel) where the first click has a substantially higher peak formant frequency. All codas are of *a*-coda vowel type.

Figure 5a shows three diphthongal and two level codas by Fork. The first four codas are consecutive. They show Fork first vocalizes two codas with an upward trajectory in formant frequencies, followed by a coda with a downward trajectory, and then a level coda. The peak frequency of the first click is slightly higher than the rest of the coda (which also constitutes a common type, see below). The first four codas (three diphthongal and one level) are of the *a*-coda vowel type. The last coda is not consecutive, but added here to represent Fork’s level *i*-coda vowel. The formant trajectories below the 10 kHz range on spectrograms in Figure 5a illustrate these diphthongal patterns. The figure also illustrates that formant trajectory can be either upward or downward on the same coda type, and is thus likely not influenced by the number of clicks or their timing. The movement and depth values show no clear differences in movement or depth between level and diphthongal codas.

Figure 5b even more strongly illustrates that depth, head, pitch, and roll do not play a crucial role in formation of diphthongs. Figure 5b features three codas from another whale, TBB: one coda with a level trajectory and two codas with substantial falling or rising trajectories in formant frequencies. The figure on the left shows a spectrogram for *a*-coda vowel 8624 with a constant formant frequency across the coda. The second and third spectrograms show a substantial trajectory in formant frequencies: falling and rising. The movement data, however, point in the opposite direction: the level coda features more head, pitch, and roll movement than the diphthong codas. The change in depth appears comparable across the three codas. It appears that the formant frequency trajectories are independent of movement of whales. It appears that these diphthongs (falling or rising trajectories in formant frequencies) are actively controlled by sperm whales’ articulators and are not an automatic consequence of movement.

Another type of diphthong that our analysis uncovers across several whales are *a*-coda vowels in which the first click has a substantially higher formant frequency than the rest of the coda. These types of codas are given in Figure 5c, d. This pattern is independently present in Atwood, Fork, Fruit Salad, and Jocasta (see also Figures S4, S9 and 5c, d for more examples of this type). This pattern is so clear that it is recoverable in one instance even from a non-focal whale.

To quantify the existence of diphthongs, we calculate the distance between spectral peaks of clicks within a coda as determined by a peak finding algorithm. The distance between spectral peaks was calculated in two ways: (1) as the mean distance in Hz of the spectral peaks of each non-initial click from the corresponding peak in the first click (*c1dist*); and (2) as the mean distance in Hz of the spectral peaks of each non-initial click from the preceding click (*cndist*).

Figure 4c, d shows that most clicks in each coda show little to no change in their spectral peaks over the course of the coda. In this figure the counts of hand-labeled *a*-type codas are shown for different *c1dist* and *cndist* for the first spectral peak. Both measures show that the first spectral peak of almost 70% of these clicks have spectral peaks at or near 0 Hz from the first peak of the first click or the preceding clicks (averaged). While the majority of codas are level codas with little peak movement, there is a well-pronounced second distribution where there is a substantial spectral movement.

We analyzed spectral distances with Gaussian finite mixture models with a number of mixture component from 1 to 5. The mixture model with two components has the best Bayesian Information Criteria (BIC) for both *c1dist* and *cndist* and both in hand-annotated and automatically annotated coda vowel data. The two clusters of spectral distances suggest that diphthongs and vowels might be a distinct and controlled category in sperm whales.

### Controlling for Depth, Movement, and Hydrophone Placement

In principle, the observed spectral properties of sperm whale clicks could be attributed to hydrophone placement, artefacts in underwater acoustics, or whale movement. Here, we present evidence suggesting that the patterns we describe are not artefacts, but are probably actively controlled by sperm whales. Hydrophone placement and whale movement (towards or away from the hydrophone) have been shown to modulate spectral information in odontocetes (Branstetter et al., 2012; Miller, 2002). All our observations are made on focal whales only, i.e., the hydrophone is largely equidistant from the source throughout the recording for each whale. We show that head and roll have no effect on spectral peaks. Pitch has a weak correlation with spectral peaks. Depth has a correlation with spectral peak, but we show that all described patterns are possible at different depths/depth trajectories and provide several pieces of evidence suggesting that vowel and diphthong patterns are not crucially affected by depth. Additionally, a simultaneous recording of a set of clicks from two whales suggest that hydrophone placement does not crucially alter our described patterns.

Our analysis suggests that the described patterns are not automatic artefacts of whale movement. To test whether diphthongal patterns or spectral patterns in general are an automatic consequence of whale movement (i.e., correlate with whale movements) or are controlled by whales irrespective of their movement patterns, we perform a correlation test between depth, head, pitch, and roll recording from tags and spectral peaks in their vocalizations. Welch spectra were extracted with the *scipy.signal.welch* function and correlated to movement data using the Pearson correlation test. For all correlation analysis, we removed Sally’s and Jocasta’s codas because Sally’s recording includes TBB’s codas and because Jocasta’s codas were in part temporally misaligned. Head and roll have no correlation with spectral peaks: (*r* = 0.002 for head, *r* = 0.01 for roll). Pitch has a weak inverse correlation (*r* = −0.26) and depth has a correlation of *r* = 0.59 with spectral peak. That pitch and depth values are correlated with the spectral peak is not surprising as the volume of the air sac is expected to decrease as the whale dives. Importantly, as is shown throughout this paper, both diphthongal patterns and the *a*-/*i*-coda vowels are made at various pitches and depths or depth changes. For example, diphthongs like the ones produced by Fork in Figure 5a or codas with substantially higher first click (Figures 5c, d, S4, and S9 in the Supplementary Materials) can happen close to the water surface with very little depth movement of the whale.

Because depth is the only parameter that shows moderate correlation with spectral peak, we primarily focus on showing that all described patterns can be produced at different depths. We also show other parameters (head, pitch, roll) do not affect the patterns (Figures 5a and 5b), but to a lesser degree than depth because the correlation tests show less correlation with these other parameters.

Additionally, if depth crucially affected formant frequencies, we would expect to see unidirectional diphthongs when depth increases or decreases. The diphthongal patterns in Tweak (the rising-falling pattern in Figure 5c) and especially the pattern observed in TBB point to the contrary. For example, Figure 5b shows codas of TBB when she was diving. The penult coda has a substantial falling pattern, while the ultimate coda has a substantial rising pattern (the antepenult coda has the flat pattern), despite TBB moderately diving throughout these codas.

We have evidence suggesting that hydrophone placement on the whale, and therefore relative orientation of their nasal complex to the hydrophone, does not critically affect the described spectral properties either. The best piece of evidence suggesting this comes from a recording session when two whales, Sally and TBB, were both tagged. The recordings on Sally’s tag match TBB’s tag almost fully, which suggests that hydrophone placement does not crucially affect the spectrum and that the coda vowels are observable in recordings made by a tag on a whale which did not produce them. (For details, see Supplementary Materials Section 3.)

### Dialogue

Sperm whales are known to engage in dialogue with each other, in which individuals exchange codas, of varying types, in sequence (Schulz et al., 2008). This often results in overlapping codas, where two whales produce codas at the same time, frequently by making codas of the same type within milliseconds (the so-called echo-codas; Weilgart, 1990; Whitehead, 2003). The overlapping codas have been studied in detail, primarily from the perspective of timing and coda types: it appears that whales start converging on coda types and timing during dialogues (Schulz et al., 2008); as well as matching the duration and changes in duration of each other’s codas during exchanges (Sharma et al., 2024). To our knowledge, no spectral analyses of dialogues have been conducted so far.

Our new approach is useful for analyzing these dialogues. If a non-focal whale is in close proximity to a focal whale, we can observe the use of *a*- and *i*-coda vowels in both whales. Figures 3a and S5 feature codas from two bouts of Pinchy and another whale (Figure S5) who was not wearing a tag. Table 3 indicates which codas in Figures 3a and S5 are overlapping. We observe that what traditionally was analyzed as repetition of 1 + 1 + 3 codas in a dialogue is more complex when the spectral features are analyzed: both *a*- and *i*-coda vowels are observed in both the focal whale “Pinchy” and the corresponding non-focal whale.

**Table 3.** A dialogue between Pinchy and a non-focal whale from two bouts (divided by a horizontal line). If two codas are less than 1 s apart, they are transcribed in the same line. In the second bout, two codas (unmarked here) are probably from a third whale.

Pinchy #	Non-focal #	Pinchy Type	Non-focal Type	Pinchy Vowel	Non-focal Vowel
6893		9i		i	
6894		9i		i	
6895	6915	5R2	5R2	i	a
6896	6916	5R2	5R2	i	i
6897		5R2		i	
6898		1 + 1 + 3		i	
	6917		1 + 1 + 3		a
	6918		1 + 1 + 3		i
6899	6919	1 + 1 + 3	1 + 1 + 3	i	i
	6920		1 + 1 + 3		i
6900	6921	1 + 1 + 3	1 + 1 + 3	a	i
6901	6922	1 + 1 + 3	1 + 1 + 3	a	i
6902	6923	1 + 1 + 3	1 + 1 + 3	a	i
6903	6924	1 + 1 + 3	1 + 1 + 3	a	a
6904		1 + 1 + 3		a	
	6925		1 + 1 + 3	i	
	6926		1 + 1 + 3	i	
6905		1 + 1 + 3		i	
6906		1 + 1 + 3		i	
6907		1 + 1 + 3		i	
6908		1 + 1 + 3		i	
6909		1 + 1 + 3		i	
6910	6927	1 + 1 + 3	1 + 1 + 3	i	i
6911	6928	1 + 1 + 3	1 + 1 + 3	i	i
6912		1 + 1 + 3		a	
6913	6929	1 + 1 + 3	1 + 1 + 3	a	i
6914		1 + 1 + 3		i	

6930	6938	1 + 1 + 3	1 + 1 + 3	a	a
6931		1 + 1 + 3		a	
6932		1 + 1 + 3		i	
6933		1 + 1 + 3		i	
6934		1 + 1 + 3		i	
6935		1 + 1 + 3		i	
6936	6939	1 + 1 + 3	1 + 1 + 3	i	i
6937		1 + 1 + 3		a	
6940	6959	1 + 1 + 3	1 + 1 + 3	a	i
6941	6960	1 + 1 + 3	1 + 1 + 3	a	i
6942	6961	1 + 1 + 3	1 + 1 + 3	a	a
6943	6962	1 + 1 + 3	1 + 1 + 3	a	a
6944	6963	1 + 1 + 3	1 + 1 + 3	?	i
6945	6964	1 + 1 + 3	1 + 1 + 3	i	i
	6965		1 + 1 + 3		i?
	6966		1 + 1 + 3		i
	6967		1 + 1 + 3		i
6946		1 + 1 + 3		a	
6947		1 + 1 + 3		i	
6948		1 + 1 + 3		i	
6949		1 + 1 + 3		i	
6950		1 + 1 + 3		i	
6951		1 + 1 + 3		i	
6952		1 + 1 + 3		a	
6953		1 + 1 + 3		a	
6954		1 + 1 + 3		a	
6955		1 + 1 + 3		a	
6956		1 + 1 + 3		i	
6957		1 + 1 + 3		i	
6958		7i		i	

Table 3 annotates these two bouts of dialogue between Pinchy and her interlocutor in terms of the *a*- and *i*-coda vowels. We annotate them for the two proposed types (annotations can be verified by the spectrograms in Figures 3a and S5). Another similar dialogue is observed on two non-focal whales (Table S1 and Figure S6) when Fork was wearing the tag.

If only traditional coda types were analyzed, this dialogue would appear as a simple repetition of coda types. With the analysis of vowels on codas, it appears that the whales are vocalizing the two different elements (*a* and *i*). When two whales are vocalizing simultaneously (overlapping codas), and when producing the same coda rhythm type (1 + 1 + 3 in this case), they can either match the same coda vowel (*a*-*a* or *i*-*i*) or produce different coda vowels (*a*-*i*). Whether this exchange is random or follows structural patterns is left for future studies when more extensive data on dialogues might become available. Table 3 illustrates the transcription of *a*- and *i*-coda vowels in a dialogue as well as the proportion of overlapping and non-overlapping codas (coded as transcribed in the same row or a new row).

The fact that *a*- and *i*-coda vowels are visible from non-tagged whales also means that the whales can not only produce but also likely hear (and perceive) this difference. In other words, hydrophones capture most of the observed spectral properties on both focal and non-focal whales (Figure S1 in Supplementary Materials). It is likely that these properties get distorted at a distance or with movement (Miller, 2002), but the effects of distance are currently difficult to estimate. Even if underwater acoustics distorts the signal at distance substantially, it is likely that the difference between the *a*- and *i*-coda vowels have multiple spectral cues beyond the formants that we describe, which would facilitate perception of the two coda vowels. The hearing ability of sperm whales is strong in the frequency range where we observe the patterns, and it can exceed 30 kHz (Schmidt et al., 2018). The ability to detect coda vowels on non-focal whales also suggests that the observed patterns are not an artefact of hydrophone placement on the whale.

## DISCUSSION

### Articulatory Control

Our proposal suggests that spectral patterns (vocalic and diphthongal) require articulatory control in sperm whales. While there are many aspects of sperm whale articulation that are not yet fully understood, recent work has suggested that sperm whales and other odontocetes can control articulators to a larger degree than previously thought (Madsen et al., 2023). Weir et al. (2007) argues that the tonal, burst-pulse ‘squeal’ vocalization of sperm whales, which is different from coda vocalizations, might be controlled by the whales, resulting in spectral modulations of squeals. Sperm whales have also been shown to produce other types of vocalizations, such as trumpets (Pace et al., 2021), which additionally points to at least some level of active articulatory control. This line of work, however, focuses on registers that produce different kinds of vocalizations and not on differences within codas. Nevertheless, the work in Weir et al. (2007), Pace et al. (2021), and Madsen et al. (2023) suggest that active modulation of vocalizations is possible. We also know that echolocation clicks are acoustically distinct from coda clicks, which is perhaps achieved by distal air sac shape (Madsen, Payne, et al., 2002). It has also been suggested that sperm whales may be able to create conformational changes to their nasal complex that could change the distance between reflective air sacs by 10 percent through the contraction of longitudinal muscles which could pull soft parts of the nose back towards the skull (Bøttcher et al., 2018). It is possible that similar changes in the nasal complex could lead to the described changes in spectral properties of coda vowels, but articulatory predictions are currently difficult to test in sperm whales. Given that the spermaceti organ is surrounded by muscles on the sides and air sacs on the ends of the organ (Huggenberger et al., 2016), it is not impossible to assume that the whales can control changes in the resonant body that are substantial enough to result in our observed patterns.

It has long been established that the source of coda vocalizations are the phonic lips. We speculate that the distal air sac acts as a filter resulting in the observed spectral properties. Phonic lips appear at the entrance of the distal air sac (Cranford, 1999; Huggenberger et al., 2016), which is parallel to human articulators where vocal folds appear at the entrance of the vocal tract. We assume that the sound containing the described formants (which result from the distal air sac modulations) then propagate in the spermaceti organ. These propagations are reflected in the acoustics as click pulses.

In parallel to human vocalic formants, we can model the formants of coda vowels as resonant frequencies of a simple tube. Some human vowels are best approximated with a simple tube open at one end (oral opening), others are best approximated as a combination of tubes, including tubes closed at both ends (Stevens, 1999). If we model the distal air sac as a simple tube closed at both ends, the first resonant frequency can be calculated using Equation 1 (Stevens, 1999).

$$f_{n}=\frac{nc}{2L}$$ (1)

The *n*th resonant frequency (*f*_*n*_) is calculated from the velocity of sound in air (*c*, approximated at 343 m/s) and length of the simple tube (*L*). The first resonant frequency *f*_1_ will be at approximately 7827 Hz (the first peak of the first click of the diphthongal coda 6889 in Figure 5) if the length of the tube is just at 2.2 cm according to Equation 1. For the resonant frequency of approximately 6397 Hz (the first peak of the second click of the same diphthongal coda), the tube length would be just over 2.7 cm according to Equation 1. The observed spectral trajectories can thus be achieved by controlling the shape of the distal air sac by less than a centimeter. While the exact measurements of the distal air sac are lacking, analyses of sperm whale anatomy in Huggenberger et al. (2016) suggest that the length of the disk-shaped distal air sac might be within the calculated range.

### Orthogonality

The traditional sperm whale coda types appear to be at least partially independent or orthogonal to the proposed coda vowels, which means that the source features (number of pulses and timing) can be relatively independently combined with filter features (spectral properties). The *a*-coda vowel and the *i*-coda vowel can appear on 1 + 1 + 3 or 5R2 codas, for example, as do the diphthong patterns. We call the property where source features can combine with filter features the orthogonality of coda vowels. Orthogonality does not mean that the source features are completely independent of the filter features (or coda-vowel patterns). Like in human vowels, it is likely that distributional patterns exist where some filter features might be more or less common on specific source features or vice versa.

We demonstrate that sperm whale codas are composed of several relatively orthogonal or independent features. The first two features in Table 4 have previously been established. Additionally, Sharma et al. (2024) recently reported that these timing/click number properties are highly combinatorial: their established rhythm, tempo, rubato, and ornamentation have the potential to make the traditionally observed properties even more complex. Here, we propose that in addition to the number of clicks and their timing, spectral properties constitute a new set of features that can combine with already established features. In sum, the list of properties that are potentially meaningful now also includes formant patterns and formant trajectories (Table 4).

**Table 4.** A list of potentially meaningful properties.

source features:	Number of clicks
	Timing
filter features:	Formant patterns: the *a*-coda vowel and the *i*-coda vowel
	Formant trajectories: level, rising, falling diphthongs

Many of these patterns are orthogonal. We have shown that the *a*-/*i*-vowel distinction is possible on different coda rhythm and tempo types (1 + 1 + 3, 5R1, or 5R2) and diphthongal patterns can also surface on different coda rhythm types.

## CONCLUSION

This paper uncovers new patterns in sperm whale coda vocalizations and suggests that a new dimension—spectral properties of clicks in codas—might be a meaningful feature in the sperm whale communication system. Traditionally, sperm whale codas have been primarily analyzed in terms of the number of clicks and the timing between clicks. These two parameters have been used to classify codas into many traditional coda types (Weilgart & Whitehead, 1993) or recently more fine grained combinatorial timing/click features (Sharma et al., 2024).

Human spoken language employs acoustic properties to convey meaning. Vowels in human speech can differ in length (or the number of vocal pulses), timing between pulses (or the fundamental frequency F0), formant frequencies (or the quality of vowels such as [a] vs. [i]) and trajectory of vowels (monophthongs like [i] and diphthongs like [ai]).

It appears that sperm whale codas feature analogs to all these characteristics. The number of clicks and their inter-click timing can be understood as the duration of the coda and the fundamental frequency (F0). In human vowels, F0 is a dimension determined by the rate of vocal fold vibrations. In other words, the timing between vocalic pulses determines F0. In codas, the F0 is thus analogous to the timing between clicks (pulses). Human and coda vowels can also have different durations. The total duration of a human vowel is determined by the number of vocalic pulses and their individual timing. Similarly, the total duration of a coda is determined by the number of clicks and the ICIs. We do not doubt that these two timing properties constitute distinct elements in sperm whale vocalizations, as has been previously proposed. In human speech, the fundamental frequency (F0) and duration of vowels carry meaning-distinguishing information. For example, the four Mandarin tones can change the meaning of segmentally the same syllable: *mā* ‘mother’, *má* ‘hemp’, *mǎ* ‘horse’, and *mà* ‘scold’ (Duanmu, 2007). Conversely, two words can have the same tone, but a different vowel and a different meaning. While *má* means ‘hemp’, *mí* can mean ‘be lost’.

This paper suggests that, in addition to the number of clicks and ICI, resonant frequencies (formants) in sperm whale codas and their trajectories are potentially meaningful as well. We uncover formant patterns in sperm whale codas and describe at least two clear patterns: the *a*-type and the *i*-type coda vowels. We also show that the two coda vowels are discretely distributed across codas and can be uttered in sperm whale dialogues. Finally, we demonstrate that individual codas can also have rising and falling trajectories (or a combination of the two), a pattern that we call *coda diphthongs*. These patterns are likely not artefacts resulting from whales’ movement or depth position. These patterns are likely recurrent across whales, controlled by whales, perceivable, and discrete (in the sense that a coda is either of one type or the other).

Exploration of the acoustic properties in codas was prompted by a deep neural network architecture called fiwGAN (Beguš, 2021a). When fiwGAN is trained on human speech, it reliably discovers meaningful properties in human language in a fully unsupervised manner. The model has been shown to learn various degrees of complex structure in language from sounds of language (Beguš & Zhou, 2022), words (Beguš, 2021a), to complex morphophonological processes (Beguš, 2021b) in a neurally plausible way (Beguš, Zhou, & Zhao, 2023). The model, for example, learns to encode individual words into the single units in the latent space (Beguš, 2021a), suggesting that the model discovers word classes exclusively from the acoustic signal in a fully unsupervised manner. In other words, the model reliably uncovers meaningful properties in human language, where the ground truth about what is meaningful is available.

To test fiwGAN’s utility on non-human communication systems for which no ground truth exists, the network was trained to imitate sperm whale codas and embed information into these vocalizations. An interpretability technique called CDEV (Beguš, Leban, & Gero, 2023) suggested that several spectral properties might be meaningful in this communication system. By uncovering recurrent, discretely distributed patterns in spectral properties that repeat across whales and appear to be actively controlled by the whales, we make explicit the possibility that spectral properties are meaningful, as suggested by the fiwGAN model (Beguš, 2021a) and the CDEV method in Beguš, Leban, and Gero (2023). This paper thus constitutes a case where an AI interpretability technique predicted a previously unknown fact that was later confirmed with analytical acoustic tools. The advantage of AI models (such as fiwGAN) is that they are not biased by human biology and as such are able to uncover patterns that humans might have missed. We expect that the ability to analyze the internal representations of AI models (AI interpretability) will provide further insights in various fields, both in bioacoustics and beyond.

These findings have the potential to add to the communicative complexity of sperm whale vocalizations and open up several new possibilities for research. Our paper suggests that the sperm whale communication system is not exactly a Morse code-like system, but one where the spectral properties of codas are acoustically differentiated. How the spectral properties of codas are realized in other clans and how they relate to referential meaning is a future direction for this research.

## METHODS

### Data

The data stems from following and recording social units of female and immature sperm whales along the western coast of the Island of Dominica between 2005 and 2018 (details in the Appendix). This resulted in a dataset of 3948 focal and non-focal codas that were annotated as described in Supplementary Materials Section 1, maintaining temporal ordering and associating speaker identities. To explore the hypothesis that spectral properties carry meaning in sperm whales, the analysis needs to be as controlled as possible for potential underwater acoustic effects or sperm whale movement. All analyzed data are from a single channel (the first, i.e., left) from hydrophones on tags limited to only vocalizations of the focal, tagged, whales (except when explicitly stated otherwise with regards to dialogue data where we also analyze the non-focal whale engaged in a dialogue with the focal whale). This means that the hydrophone is equidistant from the whale throughout the recording period. Most of our analysis focuses on the band between 0 and 15 kHz.

Establishing the sperm whale coda vowel patterns from underwater hydrophones that are not placed directly on the whale through a tag would pose a substantial challenge, as underwater acoustics can distort the signal substantially and the apparent patterns might be attributed to spectral disturbances in the environment. For this reason, we only analyze data from hydrophones placed directly on the vocalizing whale. We have analyzed one of the largest datasets of tagged sperm whales recorded in Dominica from 2014 to 2018 by The Dominica Sperm Whale Project (Gero et al., 2014).

### Visualization

We propose a new visualization technique for the codas which removes the inter-click timing and represents spectral trends throughout the codas. To achieve this, we extract audio segments from the beginning of each annotated click and concatenate all click segments from the same coda produced by a given whale. This visualization technique allows us to remove the effects of ICI and visually observe patterns in spectral properties that would be obscured if timing were left in the visualization. For example, Figure 2 illustrates the effect of our visualization technique on the ability to find patterns in the spectral structure of codas. Two codas by tagged whale Pinchy (6931 and 6932) are visualized in waveforms and spectrograms with timing between clicks preserved on the left. On the right of the figure are two spectrograms where timing has been removed and the spectrograms are limited to the 0–15 kHz interval.

All spectrograms in the figures have a window length of 5 ms, except the spectrograms in Figure 2 which have a window length of 10 ms. The spectrogram values figures are produced in Praat software (Boersma & Weenink, 2015). Our acoustically analyzed corpus includes 7,181 coda clicks from 1,375 focal codas.

We checked for the effect of the tag/hydrophone placement on the spectral patterns by examining a subset of codas (*n* = 17) that were recorded by two tags, one on the focal whale and one on a non-focal whale (see Controlling for Depth, Movement, and Hydrophone Placement section). We further examined the effect of depth on the spectral patterns by using the tag’s pressure sensor and contrasted this with the formant frequencies in the codas.

### Time-Insensitive Coda Assembly and Type Annotation

Audio segments for every click in each coda were extracted, DC-centered, and peak-normalized within the range [−1 1]. Each extracted segment was 15 ms and was selected to start approximately 2 ms prior to the highest peak in the click waveform. The start times are approximate, since the annotated click times were not entirely consistent with respect to the location of the highest click peak. The segments were then concatenated in sequence to form what we call time-insensitive codas, since the natural timing between the clicks was removed.

The time-insensitive codas were assembled into a single waveform with a 25 ms buffer of silence added between codas. This waveform was loaded into Praat for manual analysis and labeled by one of the authors. Spectrograms of each coda were produced using a 5 ms analysis window and labeled as either *a*-type or *i*-type based on the number of spectral peaks found in the spectrogram. These labels established a baseline for the evaluation of the automatic assignment of codas into types.

### Formant Analysis

Formant analysis was applied to each time-insensitive coda separately and multiple times using several hyperparameters. Time-insensitive codas were assembled as described in Time-Insensitive Coda Assembly and Type Annotation section, except the start time and the duration of the extracted click segments were allowed to vary. Two new parameters were added to control: (1) the start time relative to the maximum value of the click peak (−2.0 ms, −1.5 ms); and (2) the durations of the extracted audio segments (3.0 ms, 3.5 ms, 4.0 ms, 4.5 ms, 5.0 ms, 14.5 ms, 15.0 ms). Time-insensitive codas were thus created by concatenating the parameterized audio segments of each of a coda’s clicks. The overall duration of the time-insensitive codas depended on the number of clicks and the duration hyperparameter value. For example, the clicks from a five-click source coda were assembled into time-insensitive codas of total durations (15.0 ms, 17.5 ms, 20.0 ms, 22.5 ms, 25.0 ms, 72.5 ms, 75.0 ms) for the varying values of the duration hyperparameter.

Analysis software commonly used in the phonetic description of human speech was used to perform formant analysis of the constructed codas. We used the Praat ‘To FormantPath …’ (TFP) function, as exposed by the Parselmouth (Jadoul et al., 2018) Python interface, to perform LPC analysis and automatically extract one or two formant tracks for each constructed coda. The TFP function provides an algorithm to ‘automatically [find] the smoothest formant trajectories for short segments of speech,’ (Weenink, 2015) a task that closely matches our constructed codas. This method creates a number of candidate formant analyses and calculates a smoothness value to select the candidate that provides the best formant values for each analysis frame. The optimal values for each analysis frame can include only the first formant (F1) or they can include both the F1 and the second formant (F2).

The hyperparameters used to control the TFP function are described in Supplementary Materials Section 4.

Formant tracking created a set of F1 and F2 values for every analysis frame in each coda. In some analysis frames the algorithm did not yield an F2 value, and in others both F1 and F2 were calculated. We classified a coda as *i*-type if every analysis frame in the coda included values for both F1 and F2 and *a*-type if F2 was not present in any of the frames.

## ACKNOWLEDGMENTS

This study was funded by Project CETI via grants from Dalio Philanthropies and Ocean X; Sea Grape Foundation; Rosamund Zander/Hansjorg Wyss through The Audacious Project: a collaborative funding initiative housed at TED. We thank David Gruber, Shafi Goldwasser, Michael Bronstein, Orr Paradise, Pratyusha Sharma, Antonio Torralba, Jacob Andreas, Daniela Rus, Giovanni Petri, Roee Diamant, and the rest of the Project CETI team for their comments.

Fieldwork for The Dominica Sperm Whale Project during tagging years in 2014–2018 was supported by a FNU fellowship for the Danish Council for Independent Research supplemented by a Sapere Aude Research Talent Award, a Carlsberg Foundation expedition grant, a grant from Focused on Nature, two Explorer Grants from the National Geographic Society, and supplementary grants from the Arizona Center for Nature Conservation, Quarters For Conservation, the Dansk Akustisks Selskab, Oticon Foundation, and the Dansk Tennis Fond all to SG. Further funding was provided by a Discovery and Equipment grants from the Natural Sciences and Engineering Research Council of Canada (NSERC) to Hal Whitehead of Dalhousie University and a FNU large frame grant and a Villum Foundation Grant to Peter Madsen of Aarhus University. We are grateful to Kristian Beedholm for CodaSorter, as well as Mark Johnson and Peter L. Tyack for their in-kind contribution of Dtags and associated code during the research. We thank the Chief Fisheries Officers and the Dominica Fisheries Division officers for research permits and their collaboration in data collection; all the crews of R/V Balaena and The DSWP team for data collection, curation, and annotation; as well as Dive Dominica, Al Dive, and W.E.T. Dominica for logistical support while in Dominica.

## AUTHOR CONTRIBUTIONS

The pattern of coda vowels and diphthongs was discovered and established by G.B. G.B.: Conceptualization; Formal analysis; Investigation; Methodology; Software; Supervision; Validation; Visualization, Writing – original draft. R.S.: Investigation; Software. A.L.: Software. M.S.: Investigation (exploratory analysis of click-level acoustics). S.G.: Data curation; Resources; Software.

## DATA AVAILABILITY STATEMENT

The data and the code for the analysis and figures is available at https://doi.org/10.17605/OSF.IO/A32KE.

## Supplementary Material


