# Supplementary material for: Vowel- and Diphthong-Like Spectral Patterns in Sperm Whale Codas
Source: Open Mind (Camb). 2025 Nov 2;9:1849–74. doi: 10.1162/OPMI.a.252 (PMC12594577; doi:10.1162/OPMI.a.252)
Supplement: Supplementary file 1 [file opmi-09-1849-s001.pdf]

Supplementary Materials for *Vowels and Diphthongs in Sperm Whales*

Gašper Beguš[1,6*], Ronald L. Sprouse[1,6], Andrej Leban[2,6], Miles Silva[3,6], and Shane Gero[4,5,6]

[1]Department of Linguistics, University of California, Berkeley, United States
[2]Department of Statistics, University of Michigan, Ann Arbor, United States
[3]Department of Brain and Cognitive Sciences, MIT, Cambridge, United States
[4]Department of Biology, Carleton University, Ottawa, Ontario, Canada
[5]The Dominica Sperm Whale Project, Roseau, Dominica
[6]Project CETI, New York, United States and Roseau, Dominica
[*]First and corresponding author: begus@berkeley.edu

ABSTRACT

This document contains data visualizations and other Supplementary Materials for the paper *Vowels and Diphthongs in Sperm Whales*.

Non-focal 1 #	Non-focal 2 #	Non-focal 1 Type	Non-focal 2 Type	Non-focal 1 Vowel	Non-focal 2 Vowel
7255	7268	1+1+3	1+1+3	a	i
7256	7269	1+1+3	1+1+3	i	i
7257	7270	1+1+3	7-noise	i	i
7258	7271	1+1+3	1+1+3	i	i
7259	7272	1+1+3	1+1+3	i	i
7260	7273	1+1+3	1+1+3	a	i
7261	7274	1+1+3	1+1+3	a	i
7262	7275	1+1+3	1+1+3	a	i
7263	7276	1+1+3	1+1+3	i	i
	7277		1+1+3		a
	7278		1+1+3		a
	7279		1+1+3		i
7264	7280	1+1+3	1+1+3	a	i
	7281		1+1+3		a
7265	7282	1+1+3	1+1+3	i	a
7266	7283	1+1+3	1+1+3	a	a
7267	7283	1+1+3		a	

Table S1. A dialogue between two non-focal whales when Fork was wearing a tag. If two codas are less than 1 s apart, they are marked as concurrent.

1 Methods

The following paragraphs in this section are not original to our paper, but taken from several other papers authored by S.G. with only minimal paraphrasing and a few additions. These paragraphs provide only facts about data collection that are important for analyzing our work but not in any way crucial to our original argument. Because the paragraphs primarily describe facts about data collection, we believe reproducing the text with this acknowledgment is more appropriate than paraphrasing the facts with different frames and presenting the work as original.

> Well-known social units sperm whales were tracked along the western coast of the Island of Dominica (N15.30 W61.40) between 2014 and 2018. Codas were recorded through the deployment of animal-borne sound and movement tags (DTag generation 3; Johnson and Tyack 2003). Tagging was accomplished on an 11-meter rigid-hulled inflatable boat using a hand pole. DTags record two-channel audio at 120 kHz or 125 kHz with a 16-bit resolution, providing a flat (±2 dB) frequency response between 0.4 and 45 kHz. Pressure and acceleration were sampled at a rate of 500 Hz with a 16-bit resolution, and were decimated to 25 Hz for analysis.
>
> Whales, including the tagged whales, were photographically identified (Arnbom, 1987). Only tag deployments from one of the two sympatric clans (EC1, the Eastern Caribbean Clan) were included in the analysis to control for any differences in repertoires between vocal clans (Gero et al., 2016a).
>
> To define codas, absolute inter-click intervals were measured as in Gero et al. (2016b), using Coda Sorter, a custom-written tool (K. Beedholm, Marine Bioacoustics Lab, Aarhus University) in LabView (National Instruments, TX, USA). Determining if codas were produced by the tagged whales or non-focal animals was accomplished in CodaSorter using estimates for each click for the angle of arrival, channel delay, centroid frequency, and inter-pulse interval (IPI, the time between the onset of the first pulse and the onset of the next pulse in the multi-pulse structure of sperm whales clicks; Møhl et al. 2003). It is possible that some clicks are misclassified as focal or non-focal, we believe the error rate is minor enough such that final conclusions of this paper are not affected. Photo-identification supported this process by identifying which whales were present and associated with the tagged whales at each surfacing. During annotation, rare, long codas were excluded from analysis (greater than 10 clicks, less than 5% of all codas recorded).

The whales in our analyzed data have the following classification numbers: Atwood (whale #5586), Fork (whale #5151), Jocasta (whale #5987), Lady Oracle (whale #5712), Nalgene (whale #5133), Pinchy (whale #5560), Sally (whale #6052), Sam (whale #5726) , Soursop (whale #5719), TBB (whale #5759), Tweak (whale #6070),

2 Data Figures

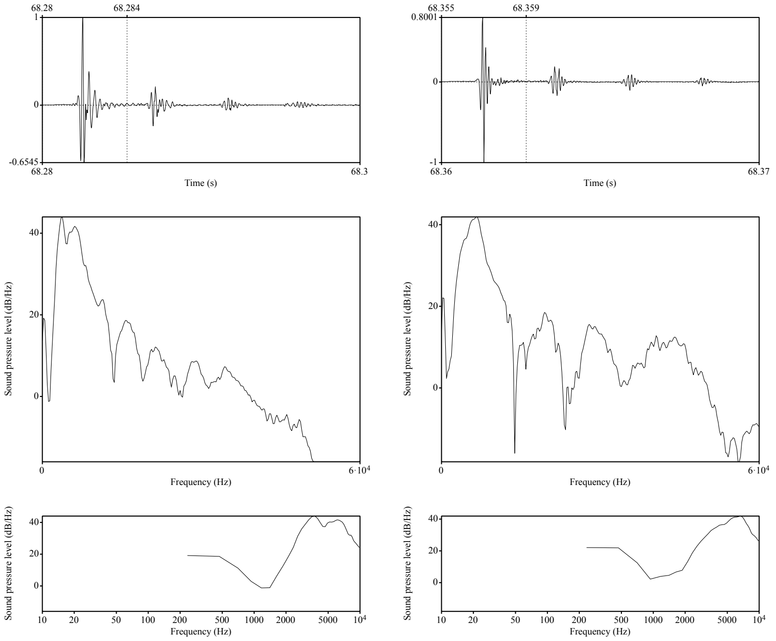

Figure S1. Waveforms and spectra (4ms window) of the first pulse of the first clicks of two codas, (**left**) one with the characteristic *i*-coda vowel pattern (coda 6911) and (**right**) a coda (coda 6912) with the characteristic *a*-coda vowel pattern. Both codas were produced consecutively by the same whale, ‘Pinchy’ (whale #5560), during the same bout. The bottom two figures show two log spectra in the 10-10,000 Hz range. Similar click-level visualizations have been made in Thode et al. (2002) and Lin et al. (2017), but the differences were ascribed to depth in Thode et al. (2002).

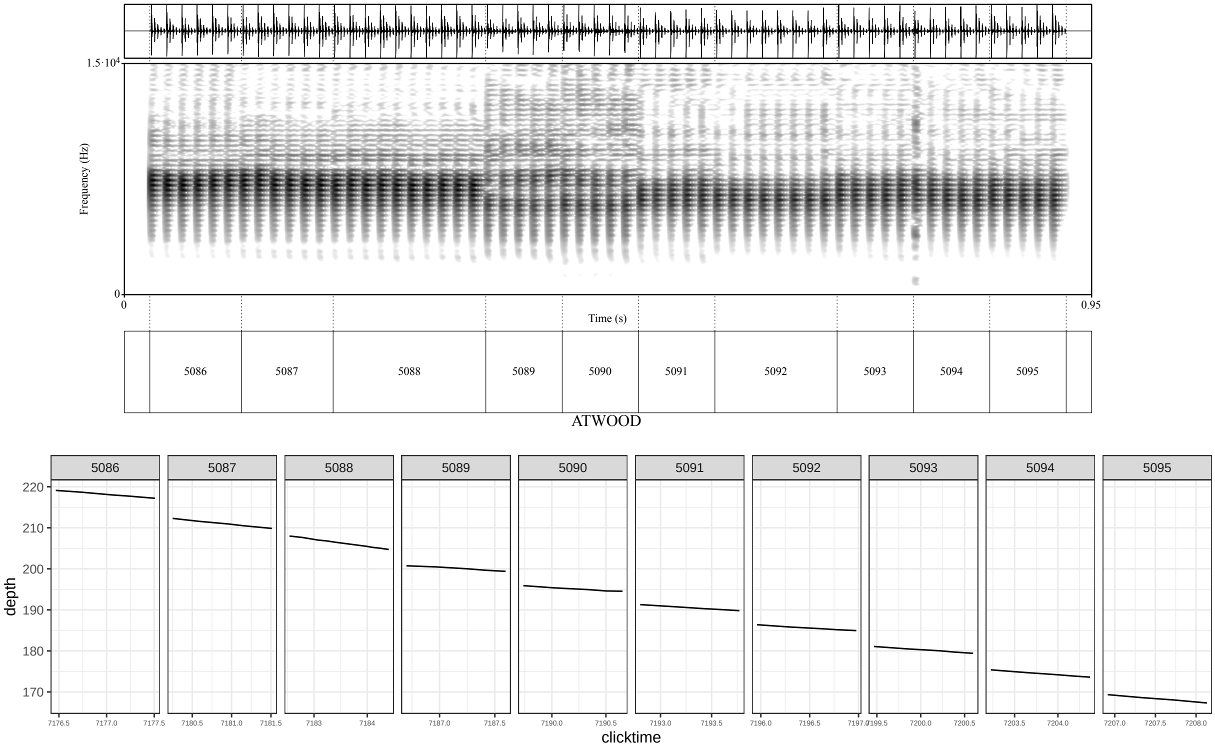

Figure S2. (**top**) Waveforms and spectrograms 0–15,000 Hz (single right channel) of 10 codas from one bout by focal Atwood with timing removed and all clicks are peak-normalized. Coda types are: 6-NOISE, 6-NOISE, 10R, 1+1+3, 1+1+3, 1+1+3, 8-NOISE, 1+1+3, 1+1+3, 1+1+3. (**bottom**) Depth values (in m) for each coda.

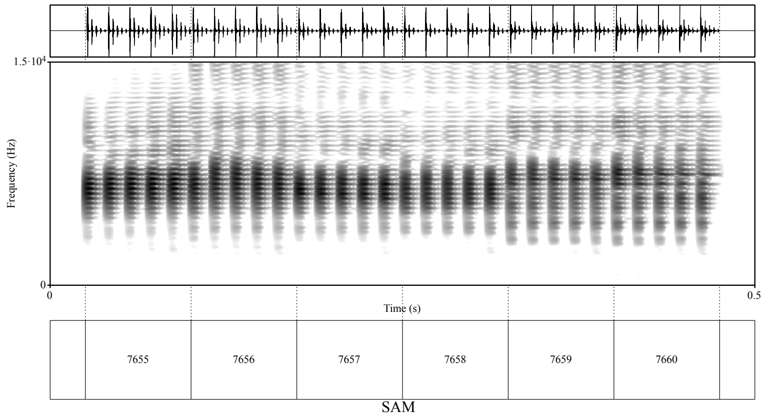

Figure S3. Waveforms and spectrograms 0–15,000 Hz (single right channel) of 6 codas from one bout by focal Sam with timing removed and all clicks are peak-normalized. All coda types are 5R1.

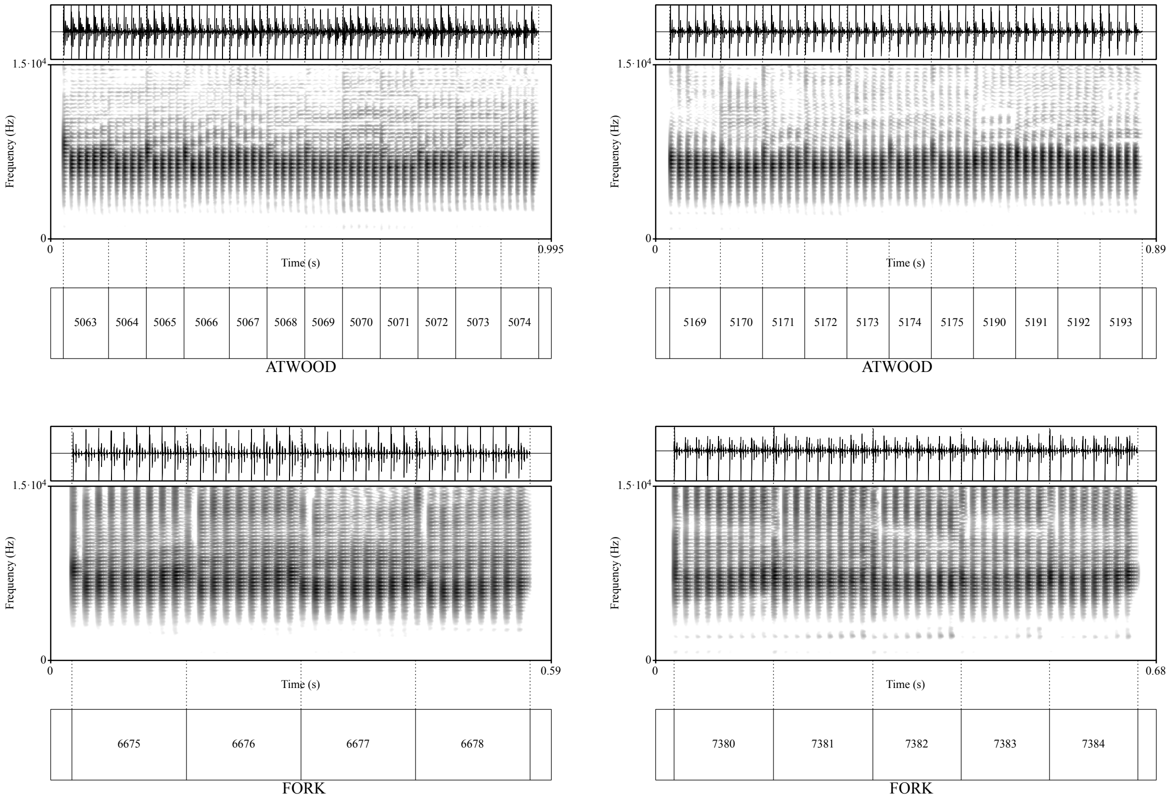

Figure S4. Waveforms and spectrograms (0–15,000 Hz, single right channel) of codas from Fork and Atwood, where the first click has a substantially higher formant frequency.

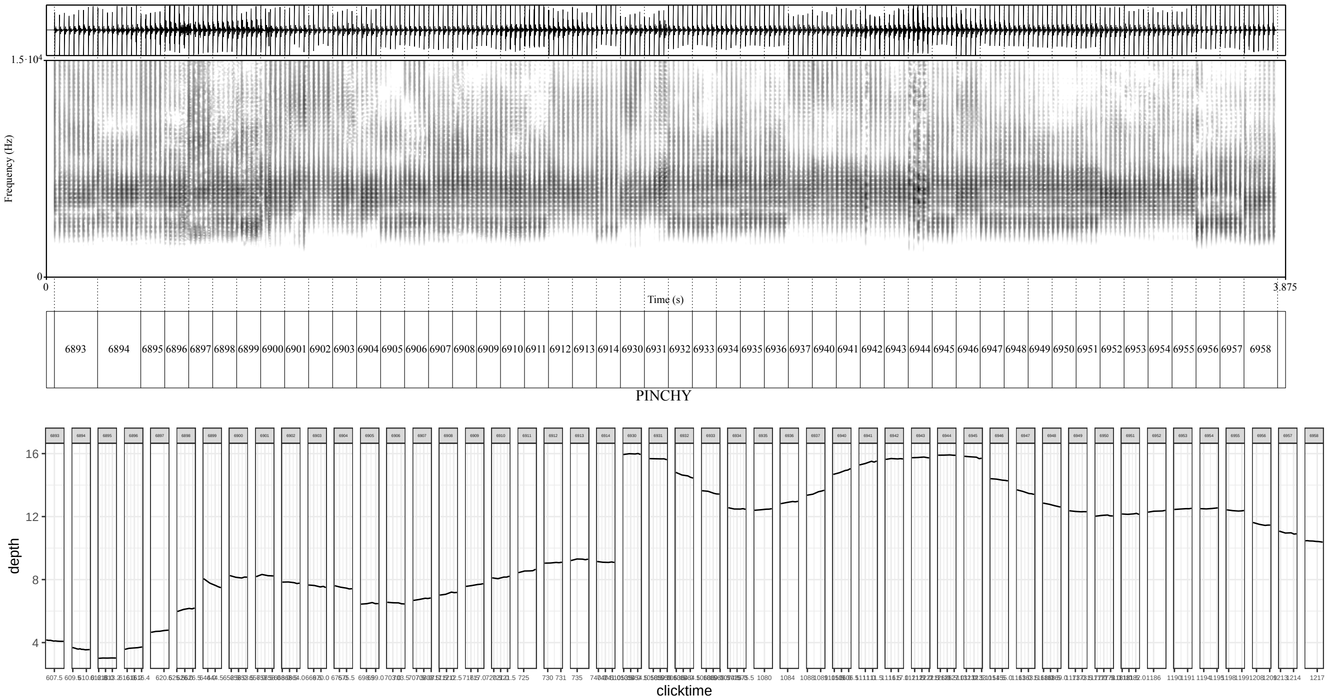

Figure S5. (**top**) Waveforms and spectrograms 0–15,000 Hz (single right channel) of Pinchy for codas analyzed in Section *Dialogue*. (**bottom**) Depth values (in m) for each coda.

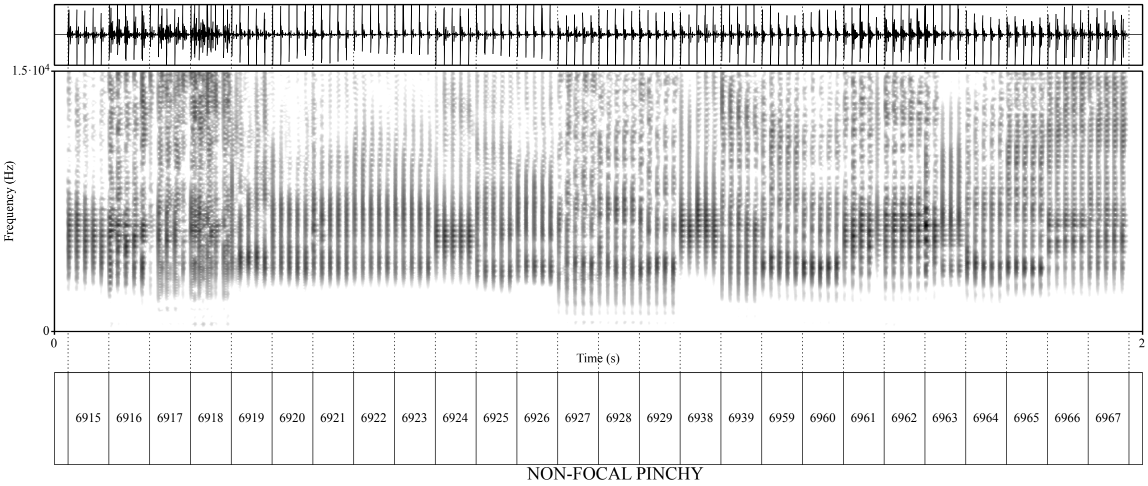

Figure S6. Waveforms and spectrograms 0–15,000 Hz (single left channel) of the non-focal whale for codas analyzed in Section *Dialogue*.

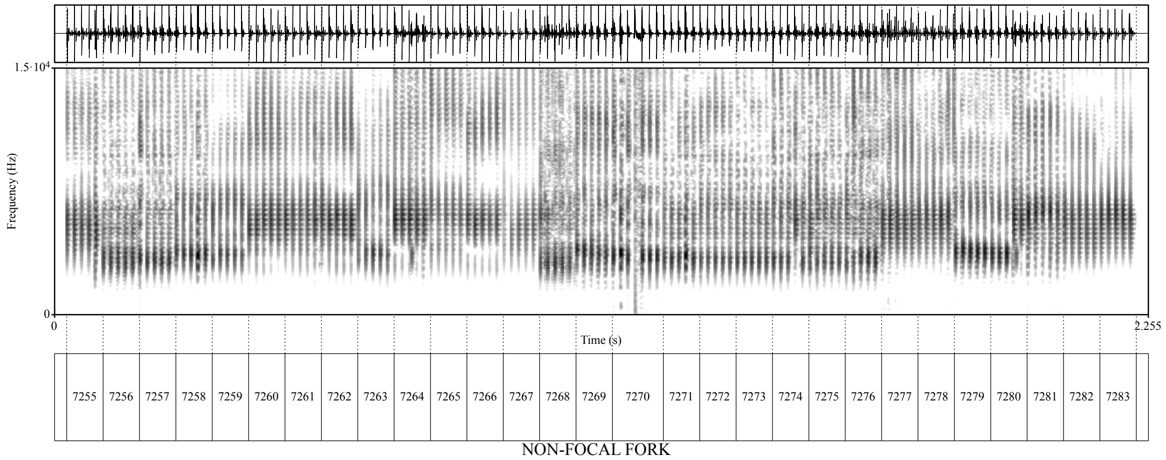

Figure S7. Waveforms and spectrograms 0–15,000 Hz (single left channel) of a dialogue in two non-focal whales analyzed in Table S1.

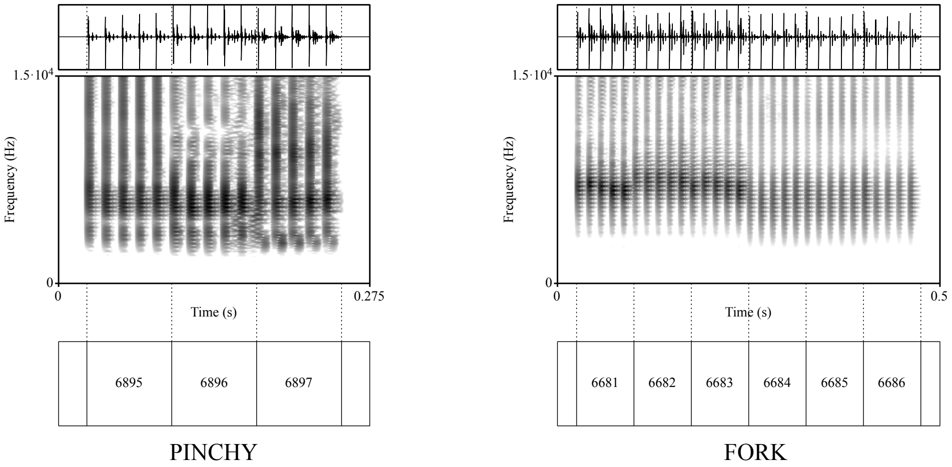

Figure S8. Waveforms and spectrograms 0–15,000 Hz (based on single, right channel) displaying the *i*-coda vowel pattern (left) by Pinchy and the *a*-coda vowel pattern (right) by Fork (whale #5151) of 5R2 codas recorded on each of their tags respectively.

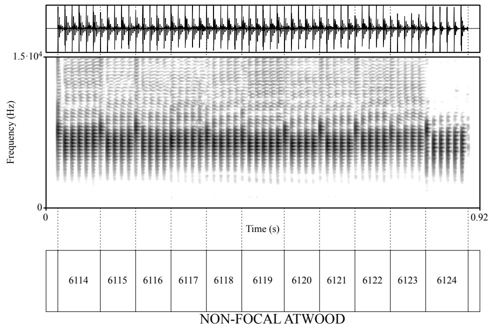

Figure S9. Waveforms and spectrograms (0–15,000 Hz, single channel) of codas from non-focal Atwood where the first click has a substantially higher peak formant frequency.

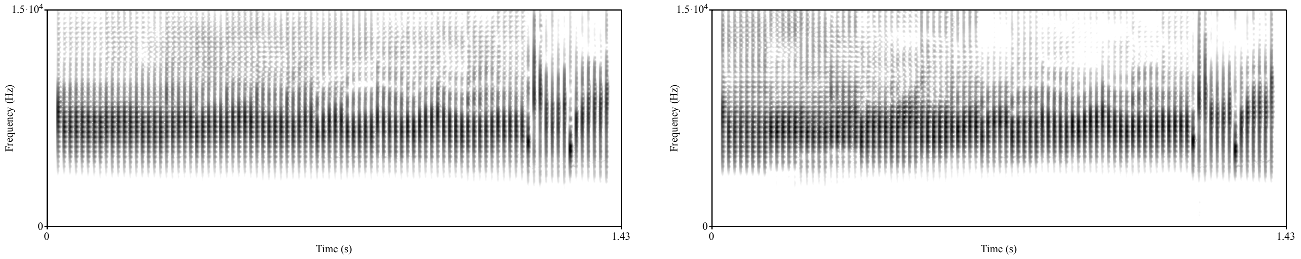

Figure S10. Spectrograms (0–15,000 Hz; single right channel) of 17 codas uttered by TBB (left) and recorded on Sally's tag (right). The left channel captures the same patterns as well, meaning that the relative position of the whale likely does not crucially affect spectral patterns when whales are in close proximity.

3 Focal and non-focal whales

Figure S10 illustrates a recording of a set of codas produced by TBB, recorded both on TBB's and Sally's tag. Judging by the amplitude of the recording, TBB and Sally were not as close in the first four codas in Figure S10, but became very close in the second half of the spectrogram in Figure S10. Sally's tag recording of TBB's vocalizations illustrates practically the exact same patterns as the TBB's actual tagged data when TBB was uttering these codas.

4 To FormantPath hyperparameters

For our analysis we parameterized the TFP function to perform 51 formant analyses to search for F1 and F2 within each time-insensitive coda, using a different maximum formant ceiling for each analysis. The formant ceilings ranged over 25 values above and below the central value of 12,000 Hz, in steps of 2 percent (7,278 Hz minimum and 19,785 Hz maximum). A non-optimal formant ceiling can result in a candidate analysis in which a spurious F2 is found when the ceiling is too high, or one in which F2 is missing when the ceiling is too low. The purpose of the 51 formant ceiling candidates is to automatically discover the analysis in which the calculated error was the smallest, thereby avoiding spurious added or missing formants.

We ran the TFP function multiple times with additional parameters to control the LPC analysis. We used the robust LPC variant (Lee, 1988; Yao et al., 2010), which provides LPC coefficient estimates with lower variance than standard LPC. The robust LPC algorithm detects and downweights residuals from standard LPC analysis, and we used three standard deviation values for the selective weighting of samples (1.0, 1.5, 2.0). The robust LPC analysis was further parameterized by selecting a variety of analysis frame sizes (3.00ms, 3.50ms, 4.00ms, 4.50ms, 5.00ms, 14.50ms, 15.00ms) and timesteps between successive analysis frames (3.00ms, 3.50ms, 4.00ms, 4.50ms, 5.00ms, 10.00ms, 12.50ms, 14.50ms, 15.00ms). Incompatible parameter combinations were excluded, such as those with a formant analysis frame duration (4.0ms) greater than the audio segment extracted per click (3.0 ms).

Finally, the TFP analysis uses polynomial functions to model the formant track alternatives and in calculating a formant candidate's smoothness criterion. Our last hyperparameter included three settings to control the polynomial order (1, 2, 3) of formant track estimation through each coda.

References

Arnbom, T. (1987). Individual identification of sperm whales. *Report of the International Whaling Commission*, 37(20):1–204.

Gero, S., Bøttcher, A., Whitehead, H., and Madsen, P. T. (2016a). Socially segregated, sympatric sperm whale clans in the Atlantic Ocean. *Royal Society Open Science*, 3(6):160061.

Gero, S., Whitehead, H., and Rendell, L. (2016b). Individual, unit and vocal clan level identity cues in sperm whale codas. *Royal Society Open Science*, 3(1):150372.

Johnson, M. and Tyack, P. L. (2003). A digital acoustic recording tag for measuring the response of wild marine mammals to sound. *IEEE Journal of Oceanic Engineering*, 28(1):3–12.

Lee, C.-H. (1988). On robust linear prediction of speech. *IEEE Trans. Acoust.*, 36(5):642–650.

Lin, C.-F., Chung, Y.-C., Zhu, J.-D., Chang, S.-H., Wen, C.-C., Parinov, I. A., and Shevtsov, S. N. (2017). The energy based characteristics of sperm whale clicks using the Hilbert Huang transform analysis method. *The Journal of the Acoustical Society of America*, 142(2):504–511.

Møhl, B., Wahlberg, M., Madsen, P. T., Heerfordt, A., and Lund, A. (2003). The monopulsed nature of sperm whale clicks. *The Journal of the Acoustical Society of America*, 114(2):1143–1154.

Thode, A., Mellinger, D. K., Stienessen, S., Martinez, A., and Mullin, K. (2002). Depth-dependent acoustic features of diving sperm whales (Physeter macrocephalus) in the Gulf of Mexico. *The Journal of the Acoustical Society of America*, 112(1):308–321.

Yao, Y., Tilsen, S., Sprouse, R. L., and Johnson, K. (2010). Automated measurement of vowel formants in the Buckeye corpus. *Gengo Kenkyu (Journal of the Linguistic Society of Japan)*, 138(0):99–113.
